# Management of Paediatric Cancers Associated With Bloom Syndrome

**DOI:** 10.1155/humu/7065233

**Published:** 2025-07-03

**Authors:** Camille Pacaud, Charlotte Nazon, Mélanie Pages, Jérémie Rouger, Pascale Berthet, Sarah Winter, Éric Thebault, Cécile Faure-Conter, Claire Berger, Catherine Paillard

**Affiliations:** ^1^Pediatric Onco-Hematology-Immunology Unit, CHU Hautepierre University Hospital, Strasbourg, France; ^2^Department of Genetics, Institut Curie, PSL Research University, Paris, France; ^3^Pediatric Oncohematology, Caen Normandy University Hospital, Caen, France; ^4^ISTCT UMR6030, OncoCARE Group, University of Caen Normandy, Normandy University, GIP CYCERON, Caen, France; ^5^Department of Cancerology, François Baclesse Center, Caen, France; ^6^SIREDO Oncology Center, Institut Curie, Paris-Saclay University (PSL), Paris, France; ^7^Pediatric Oncology and AJA Center, Lille University Hospital, Lille, France; ^8^Pediatric Hemato-Oncology Institute, Hospices Civils de Lyon, Lyon, France; ^9^Pediatric Hemato-Oncology Department, North University Hospital, Saint-Étienne, France

**Keywords:** Bloom syndrome, chromosomal breakage syndrome, paediatric cancer, toxicity, treatment modalities

## Abstract

Bloom syndrome (BS) is a rare genetic disorder associated with an elevated risk of cancer. In a national multicentre study, nine paediatric patients with BS and cancer were analysed. Median age at cancer diagnosis was 12 years. Four of the nine patients were diagnosed with BS prior to cancer detection. Six presented with solid tumours, whilst three had haematological malignancies. Six received polychemotherapy, often with dose reductions. Complications included prolonged aplasia, sepsis and early treatment discontinuation. Two patients received radiotherapy. Four relapsed, and four died, including one toxic death. However, five achieved remission, highlighting the possibility of curative treatment despite significant toxicities.

## 1. Introduction

Bloom syndrome is a rare autosomal recessive disorder caused by mutations in the BLM gene, which encodes a RecQ helicase crucial for genome stability. The condition causes genomic instability, characterised by increased rates of chromosomal breaks and mutations [1]. As of 2023, 294 cases had been registered in the Bloom Syndrome Registry (BSR), with 17 cases reported in France by late 2024 [2, 3]. Diagnosis is confirmed by molecular analysis of the BLM gene. In cases where the pathogenicity of a variant is uncertain, sister-chromatid exchange (SCE) analysis may be performed, as SCE levels are typically elevated in BS. BS presents with features such as growth deficiency, sun-sensitive skin lesions and immunodeficiency [4]. However, its most severe complication is early onset cancer, due to the underlying genetic instability [4]. Affected individuals have an increased cancer risk, with tumours appearing earlier and often multiple [1]. In the BSR, 53% (155/290) developed neoplasms; of these, 35% had multiple cancers [1]. Early life cancers include leukaemia, lymphoma, Wilms tumour, osteosarcoma and medulloblastoma [1]. From the second decade, carcinomas predominate [1, 5]. Sugrañes et al. found that 29% of cancers occurred by age 18 [1]. Median survival in individuals with BS and cancer is 36.2 years [1].

Treating cancer in BS patients is challenging due to heightened sensitivity to DNA-damaging therapies, including alkylating agents and radiotherapy [2, 6]. Children often suffer severe toxicities, even with reduced doses. Evidence remains limited, and outcomes range from remission to relapse or treatment-related death [7–11]. Management typically involves dose adjustments, close monitoring and avoiding ionising radiation when possible [12–14]. Data sharing on cancer treatment in BS remains limited, collaborative research is scarce, therapeutic decisions are largely empirical and outcomes in this high-risk population remain poorly characterised.

This retrospective study reports on the clinical features, treatments, toxicities and outcomes of children with BS and cancer in France.

## 2. Results

### 2.1. Patients and Methods

This study included patients with a molecularly confirmed diagnosis of BS. Following a national database screening and consultation with paediatric oncologists, nine patients under 18 years of age at cancer diagnosis were identified between 1994 and 2023. Medical records were reviewed for cancer diagnosis, treatment modalities, outcomes and treatment-related toxicity. Consent was obtained from all surviving patients or their guardians, and ethical approval was granted.

Seven of the nine patients were male. The median age at diagnosis of BS was 6 years (range 2–18 years). Four patients had a known diagnosis of BS prior to cancer detection; in one of them, malignancy was discovered during routine surveillance. The median age at cancer diagnosis was 12 years (range 4–16 years). Six individuals received chemotherapy, six underwent surgery, two received radiotherapy and one underwent haematopoietic stem cell transplantation. Four patients experienced relapse, and two developed subsequent malignancies: colorectal cancer and low-grade HPV-positive cervical neoplasia (HPV + CIN). At the time of analysis, five patients were in remission, whilst four had died—three from cancer progression and one from treatment-related cardiogenic shock. The median age at death was 14.5 years (range: 6–23). The median interval from birth to initial cancer diagnosis was 5 years (range: 1–17). Cancer characteristics at diagnosis are summarised in Table 1.

### 2.2. Individual Clinical Summaries

#### 2.2.1. Patient 1

A male patient, developed B-cell ALL at 14 years, was treated according to the EORTC protocol [15], achieving a complete remission. He relapsed 8 years later with medullary and CNS involvement and received the CHEPRALL protocol [16]. Complications included febrile neutropenia, Grade IV mucositis and an unexplained encephalopathy during consolidation with epratuzumab. After achieving remission, he developed pancytopenia during maintenance therapy. A second relapse occurred 1.5 years later, treated with high-dose methotrexate and cytarabine. He underwent haploidentical HSCT with TBF conditioning. Posttransplant complications were severe, including cardiac failure, infections and sinusoidal obstruction syndrome. He died from cardiogenic shock and pneumonia 2 months posttransplant. Diagnosis of BS was postmortem.

#### 2.2.2. Patient 2

A male patient with characteristic facies and growth retardation was diagnosed with BS at the age of 5 years. He was regularly monitored and, at the age of 9 years, presented with macroscopic haematuria. Imaging revealed a left renal mass, and biopsy confirmed a Stage II SMARCB1-deficient rhabdoid tumour. He underwent total nephrectomy followed by chemotherapy adapted from the EU-RHAB protocol [17] with significant dose reductions by up to 70%. Toxicities included febrile neutropenia, prolonged aplasia and repeated treatment delays. He completed nine cycles and remains in remission under regular surveillance.

#### 2.2.3. Patient 3

A male patient was diagnosed with BS at Age 8, presenting with characteristic facies, growth deficiency, photosensitivity and recurrent ENT infections. At Age 15, he developed corticosensitive, nonhyperleukocytic B-cell ALL with *t*(1; 19) and was treated with full-dose EORTC protocol [15]. Treatment was complicated by persistent aplasia, transient steroid-induced diabetes and hepatic iron overload requiring venesection. Due to recurrent aplasia, maintenance therapy was shortened by over a year. He remained in remission until Age 22, when he developed metastatic sigmoid adenocarcinoma. He died shortly after from septic shock during palliative chemotherapy.

#### 2.2.4. Patient 4

A male patient was diagnosed with BS at the age of 6, with a history of growth retardation, microcephaly and achromic skin patches. He had been under regular follow-up since diagnosis. During a routine dermatological examination, a slightly infiltrated pigmented lesion was noted beneath the right nipple, showing rapid growth. Biopsy confirmed a dermatofibrosarcoma with PDGFB rearrangement. Complete surgical excision was achieved, and the tumour was staged as T1N0. One year after diagnosis, the patient remains in remission.

#### 2.2.5. Patient 5

A male patient with growth retardation and microcephaly was diagnosed with BS at Age 6 after SCE testing and identification of a homozygous BLM Exon 7 variant. At Age 6, he presented with cervical lymphadenopathy and general symptoms, leading to a diagnosis of ALK-negative anaplastic large-cell lymphoma, Stage II. The initial treatment according to the EURO-LB02 [18] failed to induce remission. Subsequent salvage regimens were administered without dose reduction. Despite initial response and radiotherapy, disease progression occurred, and he died 9 months after diagnosis.

#### 2.2.6. Patient 6

A female patient born prematurely with intrauterine growth restriction was diagnosed at Age 15 with osteoblastic osteosarcoma of the proximal left tibia. She was treated according to the SARCOME-09 protocol [19]. Due to poor tolerance, including prolonged aplasia, chemotherapy doses were reduced from the outset by up to 50%. Significant toxicities led to the diagnosis of BS during treatment. Three years later, she developed a bone metastasis in the left talus, refractory to neoadjuvant chemotherapy, requiring amputation due to local progression. She remains in remission, with over 10 years of follow-up since diagnosis and more than 2 years since the last treatment.

#### 2.2.7. Patient 7

A male patient with growth retardation and T-cell lymphopaenia was diagnosed with oligodendroglioma at Age 12, treated by complete surgical resection without adjuvant therapy. Considering his clinical features, BS was suspected and confirmed at Age 15, with a homozygous BLM Exon 8 deletion. He remains in remission with long-term follow-up.

#### 2.2.8. Patient 8

A male patient with growth retardation was diagnosed with BS at Age 2, confirmed by increased SCE. At Age 4, he developed a left renal mass diagnosed as nephroblastoma. He received SIOP-9 chemotherapy [20], but treatment was poorly tolerated despite dose reductions of up to 30%, with repeated bacterial sepsis and prolonged aplasia. Radiotherapy to the left renal bed (14.4 Gy) was well tolerated. He experienced two relapses: first locally, treated with chemotherapy, surgery and pelvic radiotherapy, then retrohepatic, refractory to further treatment. He died at Age 6 from peritoneal carcinomatosis. This case has previously been reported by Berger et al. [21].

#### 2.2.9. Patient 9

A female patient was diagnosed with a dermatofibrosarcoma protuberans at Age 16, treated with complete surgical excision. She remained in remission with a 5-year follow-up. At Age 18, she was diagnosed with BS through familial screening, carrying a homozygous pathogenic BLM variant. At Age 19, she was treated locally for a low-grade HPV + CIN.

## 3. Discussion

We reported nine children with BS who developed neoplasms in France between 1994 and 2023. Due to the absence of a comprehensive registry, identifying such cases is challenging [22]. BS's genetic locus was identified in 1997, meaning that reliable molecular biology registries have only been available since then. Certain types of neoplasms, such as dermatofibrosarcoma and rhabdoid tumours, have not previously been described in patients with BS [1].

This study shows that out of the nine children with BS and cancer in France, five achieved a remission, with highly variable durations due to the small cohort size and the rarity of the condition.

However, our data align with the literature regarding the acute toxicity of chemotherapy in children, with one toxic death [7–11]. It is noteworthy that all patients who achieved remission had undergone dose reductions in chemotherapy, tailored to individual tolerance. There is limited literature about HSCT and BS for children [2]. Our findings are consistent with existing literature on the acute toxicity of chemotherapy in children, including one treatment-related death. Notably, all patients who achieved remission had received reduced doses of chemotherapy, adjusted according to individual tolerance. In our experience, one patient underwent HSCT with a myeloablative conditioning regimen but died shortly after transplantation due to treatment-related toxicity, despite being in remission at the time. Although evidence is limited, achieving a balance between efficacy and toxicity remains challenging, as BS cells are highly sensitive to chemotherapy, whilst tumour cells may develop resistance [23].

Interestingly, radiotherapy was well tolerated, with no complications in the two patients treated, challenging previous assumptions about its risk in BS [4]. In the literature, the role of radiotherapy in BS remains uncertain and requires further investigation [6, 9, 24].

To our knowledge, our study represents the largest paediatric cohort to date describing treatment approaches and outcomes in patients with BS diagnosed with cancer. Broader data collection will require international collaborative studies, which is one of the key priorities for affected families [22]. Investigating the potential role of targeted therapies, such as checkpoint inhibitors and immunotherapy, in the management of this chromosomal breakage syndrome could be of significant interest, particularly considering its chemoradiosensitivity and elevated risk of secondary cancers [10, 22].

## Figures and Tables

**Table 1:** Clinical and genetic characteristics of children with BS and cancer.

	**Pt 1**	**Pt 2**	**Pt 3**	**Pt 4**	**Pt 5**	**Pt 6**	**Pt 7**	**Pt 8**	**Pt 9**
Sex	M	M	M	M	M	F	M	M	F
Cancer type(s)	B-ALL	Renal rhabdoid tumour	B-ALL, CRC (adult)	DFS	ALK-negative ALCL	Osteosarcoma	Oligodendroglioma	Nephroblastoma	DFS, HPV + CIN (adult)
Age at diagnosis (years)	14	9	15	6	6	15	12	4	16
Treatment modality	Chemotherapy, HSCT	Surgery, chemotherapy	Chemotherapy	Surgery	Chemotherapy, radiotherapy	Surgery, chemotherapy	Surgery	Chemotherapy, radiotherapy	Surgery
Outcome	Deceased	Remission	Deceased	Remission	Deceased	Remission	Remission	Deceased	Remission
Follow-up (years)	9	4	7	1	0	10	13	2	5
Treatment toxicity	Early discontinuation, prolonged aplasia, severe infections, organ failure	Prolonged aplasia	Early discontinuation, prolonged aplasia, iron overload	None reported	Early discontinuation, prolonged aplasia	Early discontinuation, prolonged aplasia, severe infections	None reported	Prolonged aplasia, severe infections	None reported
BLM testing time	Postmortem	Pretreatment	Pretreatment	Pretreatment	During treatment	During treatment	Posttreatment	Pretreatment	Posttreatment
BLM variant(s)	c.98+2dup (hom)	p.Trp506∗ (hom)	p.Phe194∗ (hom)	Exon 15 deletion (hom)	Exon 7 (double het)	p.Gln752∗ (het), p.Arg959∗ (mosaic)	Frameshift (hom, Exon 8)	Not specified	c.98+2dup (hom)

Abbreviations: ALCL, anaplastic large; ALL, B; cell, lymphoma; CIN, human papillomavirus; CRC, colorectal cancer; DFS, dermatofibrosarcoma; femal, e; het, heterozygous; hom, homozygous; HP, V; HSCT, haematopoietic stem cell transplantation; mal, e; positive, cervical intraepithelial neoplasia; Pt, patient.

## Data Availability

The data that support the findings of this study are available from the corresponding author upon reasonable request.

## Nomenclature

ALL: acute lymphoblastic leukaemia
ALK: anaplastic lymphoma kinase
BSR: Bloom Syndrome Registry
BS: Bloom syndrome
CHEPRALL: chemotherapy protocol for acute lymphoblastic leukaemia
CNS: central nervous system
DNA: deoxyribonucleic acid
EORTC: European Organisation for Research and Treatment of Cancer
EU-RHAB: European Rhabdoid Tumour Registry Protocol
EUROLB02: European Lymphoma Protocol 02
HPV: human papillomavirus
HSCT: haematopoietic stem cell transplantation
PDGFB: platelet-derived growth factor beta
SCE: sister-chromatid exchanges
SIOP-9: International Society of Paediatric Oncology Protocol 9
SMARCB1: SWI/SNF-related, matrix-associated, actin-dependent regulator of chromatin, Subfamily B, Member 1
TBF: treosulfan, busulfan, fludarabine
